# Seroprotection to five vaccine-preventable diseases among children in East New Britain, Papua New Guinea

**DOI:** 10.1016/j.lanwpc.2026.101881

**Published:** 2026-05-22

**Authors:** Stefanie Vaccher, Maria Ome-Kaius, Daisy Mantila, Damitha Rakuafery, Milena Dalton, Clare Valaki, Pele Melepia, Patrick Kiromat, Elsie Stanley, Edward Waramin, Michelle J.L. Scoullar, Moses Laman, Fiona Angrisano, Leanne J. Robinson

**Affiliations:** a Burnet Institute, 85 Commercial Rd, Melbourne, VIC 3004, Australia; b Papua New Guinea Institute of Medical Research, Goroka P.O. Box 60, Papua New Guinea; c Faculty of Medicine, Dentistry and Health Sciences, University of Melbourne, 161 Barry St, Carlton, VIC 3010, Australia; d Burnet Institute, Section 90, Lot 1 Takubar, Kokopo P.O. Box 1458, Papua New Guinea; e East New Britain Provincial Health Authority, Butuwin, Kokopo P.O. Box 714, Papua New Guinea; f Papua New Guinea National Department of Health, Aopi Building Centre, Waigani Dr, National Capital District, Papua New Guinea; g The School of Public Health and Preventive Medicine, Monash University, 553 St Kilda Road, Melbourne, VIC 3004, Australia

Vaccine coverage in Papua New Guinea (PNG) remains low and inconsistent. In 2024, WHO/UNICEF estimates ranked PNG second lowest globally for DTP1 coverage (49%) and fifth lowest for MR1 (44%).^1^ This study determined the seroprevalence of five vaccine-preventable diseases (VPDs), diphtheria, tetanus, pertussis, measles, and rubella, in children aged 10–23 months from two districts of East New Britain Province (ENBP) to identify immunity gaps.

The study was conducted in five priority local-level government (LLG) areas representing urban, rural, and remote island settings, identified based on high proportions of zero-dose (no DTP1) and under-immunised (no DTP3) children.^2^^,^^3^ As such, the study sample may modestly overrepresent caregivers who are more engaged with health services. As data on non-participants were not systematically collected, some selection bias cannot be excluded; however, any impact on observed vaccination coverage is likely minimal.

Informed written consent was obtained from parents or caregivers prior to undertaking any study procedures. Child Health Record books were reviewed, vaccination and clinical data were recorded and dried blood spot (DBS) was collected. IgG levels against the five VPDs were measured using a multiplex bead assay.^4^^,^^5^ Protective antibody levels were determined based on standards established by the World Health Organisation (WHO).^6^ Antibody concentrations were quantified using a multiplex bead assay, with values expressed in international units per millilitre (IU/mL) (Supplementary Table S1).^7^^,^^8^ Chi-squared tests were used to compare log-transformed mean fluorescent intensity (MFI) between different groups (e.g., by gender). Gender was defined based on caregiver-reported sex of the child at the time of enrolment (male or female). A total of 379 children were recruited into the study between late March and mid-August 2023, with a similar number recruited across the five target LLGs. Median age was 16 months, and 53% of participants were male (Supplementary Table S2). Vaccination data, as confirmed in the Child Health Record book, were available for 346 (91%) children, while parents/guardians of 28 children recalled vaccination for their children. However, vaccination dates could not be provided, and these 28 children were thus assumed to be partially immunised only. Breakdown of DTP and MR vaccination status is shown in Supplementary Fig. S1.

Approximately 80% of children had received at least one dose of MR-containing vaccine, including 51% (n = 194) with two recorded doses. Overall, 88% (n = 335) and 89% (n = 338) were seroprotected against measles and rubella, respectively (Supplementary Table S3). Among measles-seroprotected children, 56% had two documented MR doses and a further 31% had received at least one dose ≥28 days before sample collection No child had a prior measles or rubella diagnosis. Antibody levels and seroprotection did not differ by gender. Age was not associated with discordant protection; however, all 9 children protected against only one antigen were male (p = 0.004). Of these, two children had no record of any MR dose, and another two only had caregiver recall of vaccination. These children also appeared less likely to be seropositive for diphtheria and tetanus, although numbers were small (Fig. 1a).

**Fig. 1:**
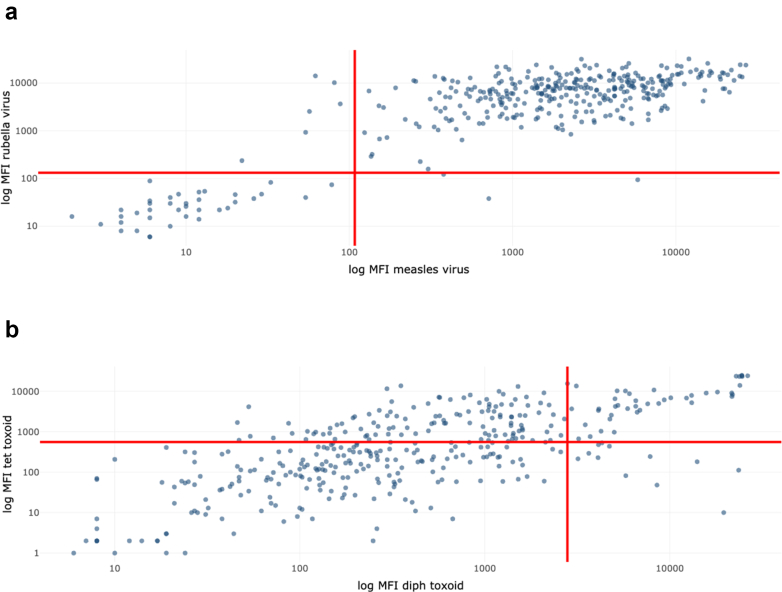
**Scatter plot of log-MFI a) anti-measles and anti-rubella IgG distribution.** Dots in the upper left or lower right quadrants indicate individuals who only displayed seroprotective antibodies against rubella (left, n = 6) or measles (right, n = 3), b) anti-diphtheria and anti-tetanus IgG. Dots in the upper left or lower right quadrants indicate individuals who only displayed seroprotective antibodies against tetanus (left, n = 125 or diphtheria (right, n = 11). Red lines indicate antibody seroprotection thresholds.

Among all children sampled, 75% (n = 285) had evidence of receiving three DTP doses (Supplementary Fig. S1). Overall, only 16% of children (n = 60) had antibody levels associated with protection against diphtheria, although 46% (n = 175) were seroprotected against tetanus. Most children (92%, n = 348) had detectable pertussis antibodies, though none had titres >100 IU/ml indicative of recent infection (Supplementary Table S3). Antibody levels and immune status did not differ significantly by sex.

Seroprotection to diphtheria was low irrespective of vaccination status (13%, 12%, and 17% in unvaccinated, partially vaccinated, and fully vaccinated children, respectively), with a similar pattern for tetanus (24%, 37%, 51%). The majority of participants had detectable pertussis antibodies (89%, 84%, 94%) (Supplementary Table S3).

Forty-five children (12%) had no documented or recalled DTP vaccination nor prior diagnosis. However, 40 (89%), 11 (24%) and 6 (13%) children had detectable antibodies to pertussis, tetanus, and diphtheria, respectively. Discrepancies may reflect incomplete records or unrecognised exposure. Among children seroprotected for tetanus but not diphtheria (n = 125) or vice versa (n = 11), age was not associated with seroprotection status. Among tetanus IgG-positive but diphtheria-negative, 10/11 were male (Fig. 1b). Overall, 81% of children with discordant diphtheria–tetanus protection had received three DTP doses (vs 75% overall). They were also less likely than the overall cohort to be seropositive for measles (p = 0.02) or rubella (p < 0.01).

In 2021, an estimated 3270 and 5446 children in ENB missed DTP1 and MR1, respectively, with 26% dropout between doses, yet caregiver surveys in the same areas reported much higher uptake (93% DTP1; 88% MCV1), highlighting limitations of administrative and recall-based data.^2^ This study provides evidence of immunity gaps among children in two districts of ENBP, PNG. While seroprotection against measles (88%) and rubella (89%) was relatively high, protection against diphtheria (16%) and tetanus (46%) was low, despite reported vaccination history. These findings, consistent with previous PNG serosurveys,^9^^,^^10^ The observed discrepancy between relatively high documented DTP vaccination coverage and low seroprotection against diphtheria and tetanus, compared with higher protection against measles and rubella, warrants careful consideration. Potential explanations include differences in antigen-specific immune responses, waning immunity for diphtheria and tetanus, variation in vaccine formulations or schedules, and differences in natural boosting or exposure patterns alongside broader health system challenges.^3^ In addition, differences in serological correlates of protection and assay performance across antigens may contribute to the observed patterns. Although limited by sample size and available data, the results emphasise the need to complement administrative coverage data with serosurveillance to better identify immunity gaps and guide booster and catch-up strategies.

In conclusion, this study provides important insights into serological profiles across multiple vaccine antigens in children in Papua New Guinea. However, given potential selection bias and the higher-than-expected vaccination coverage among participants, the findings should be interpreted within the context of these limitations. Further studies are needed to better characterise immunity in truly low-coverage populations and to identify programmatic factors influencing vaccine-derived protection.

The study received all relevant ethics approvals (Alfred Health Ethics Committee, Australia #202/22, the PNG Institute of Medical Research Institutional Review Board #2206 and the PNG Medical Research Advisory Committee #22.52).

## Contributors

**Conceptualisation and** funding **acquisition:** SV, MOK, LJR, FA, MS, EW, PK, ML, MD.

**Methodology:** FA, LJR.

**Data collection:** SV, ES, CV, PM, MD, DBS collectors.

**Supervision:** MOK, LJR.

**Formal analysis:** SV, FA, MOK, LJR.

**Antibody assays:** DM, DR, FA.

**Data interpretation and original draft:** SV, FA.

SV, MOK, LJR and FA verified the underlying data and take responsibility for its integrity. All authors reviewed and approved the final manuscript.

## Data sharing statement

All relevant data are within the paper.

## Declaration of interests

We declare no competing interests.

## Appendix A Supplementary data

Supplementary Table S3

Caption for supplementary material
