# Supplementary material for: Seroprotection to five vaccine-preventable diseases among children in East New Britain, Papua New Guinea
Source: Lancet Reg Health West Pac. 2026 May 22;70:101881. doi: 10.1016/j.lanwpc.2026.101881 (PMC13221914; doi:10.1016/j.lanwpc.2026.101881)

**Supplementary Figure 1: Diphtheria-tetanus-pertussis (DTP) and measles-rubella (MR) vaccination status (n=379).**


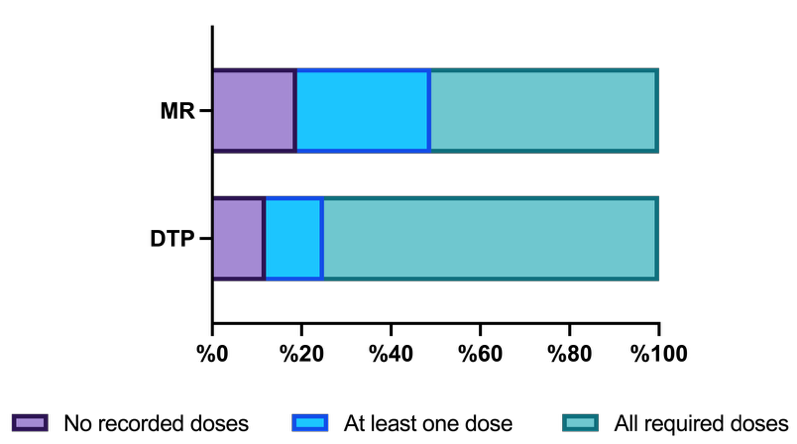

Supplement: Supplementary Fig. S1 [file mmc1.docx]
