# Supplementary material for: Seroprotection to five vaccine-preventable diseases among children in East New Britain, Papua New Guinea
Source: Lancet Reg Health West Pac. 2026 May 22;70:101881. doi: 10.1016/j.lanwpc.2026.101881 (PMC13221914; doi:10.1016/j.lanwpc.2026.101881)
Supplement: Supplementary Table S1 [file mmc2.docx]

## **Supplementary Table 1: Thresholds for seroprotection**

|  | **Susceptible** | **Partial protection** | **Full protection** |
| --- | --- | --- | --- |
| Diphtheria | ≤0.01 IU/mL | >0.01–<0.1 IU/mL | ≥0.1 IU/mL |
| Tetanus | ≤0.01 IU/mL | >0.01–<0.1 IU/mL | ≥0.1 IU/mL |
| Pertussis* | N/A | 0.8 IU/ml | >100IU/mL |
| Measles^ | N/A | N/A | 0.153 IU/mL |
| Rubella^ | N/A | N/A | 9.36 IU/mL |
