# Supplementary material for: Seroprotection to five vaccine-preventable diseases among children in East New Britain, Papua New Guinea
Source: Lancet Reg Health West Pac. 2026 May 22;70:101881. doi: 10.1016/j.lanwpc.2026.101881 (PMC13221914; doi:10.1016/j.lanwpc.2026.101881)
Supplement: Supplementary Table S2 [file mmc3.docx]

**Supplementary Table 2: Demographic characteristics of participants**

|  | **N (%)** |
| --- | --- |
| **Gender** |  |
| Male | 200 (53%) |
| Female | 179 (47%) |
| **Age (months)** |  |
| <12 | 70 (18%) |
| 12 – <18 | 169 (45%) |
| 18 – <24 | 136 (36%) |
| *Missing* | 3 (1%) |
| **Local-level government** |  |
| Bitapaka | 78 (21%) |
| Duke of York | 77 (20%) |
| Inland Baining | 66 (17%) |
| Kokopo Urban | 81 (21%) |
| Toma-Vunadidir | 77 (20%) |
