# Supplementary material for: Seroprotection to five vaccine-preventable diseases among children in East New Britain, Papua New Guinea
Source: Lancet Reg Health West Pac. 2026 May 22;70:101881. doi: 10.1016/j.lanwpc.2026.101881 (PMC13221914; doi:10.1016/j.lanwpc.2026.101881)
Supplement: Supplementary Table S3 [file mmc4.docx]

**Supplementary Table 3: Seroprotection against five vaccine preventable diseases by vaccination status (n/%)**

|  | **No vaccination** | **Partial vaccination** | **Full vaccination** | **Total** |
| --- | --- | --- | --- | --- |
| **Measles** |  |  |  |  |
| Seroprotected | 42 (58) | 104 (92) | 189 (97) | 335 (88) |
| Negative | 30 (42) | 9 (8) | 5 (3) | 44 (12) |
| **Rubella** |  |  |  |  |
| Seroprotected | 42 (58) | 107 (95) | 189 (97) | 338 (89) |
| Negative | 30 (42) | 6 (5) | 5 (3) | 41 (11) |
| **Diphtheria** |  |  |  |  |
| Seroprotected | 6 (13) | 6 (12) | 48 (17) | 60 (16) |
| Negative | 39 (87) | 43 (48) | 237 (83) | 319 (84) |
| **Tetanus** |  |  |  |  |
| Seroprotected | 11 (24) | 18 (37) | 146 (51) | 175 (46) |
| Negative | 34 (76) | 31 (63) | 139 (49) | 204 (54) |
| **Pertussis** |  |  |  |  |
| Antibodies detected | 40 (89) | 41 (84) | 267 (94) | 348 (92) |
| Negative | 5 (11) | 8 (16) | 18 (6) | 31 (8) |
