# Supplementary material for: Seroprotection to five vaccine-preventable diseases among children in East New Britain, Papua New Guinea
Source: Lancet Reg Health West Pac. 2026 May 22;70:101881. doi: 10.1016/j.lanwpc.2026.101881 (PMC13221914; doi:10.1016/j.lanwpc.2026.101881)
Supplement: Caption for supplementary material [file mmc5.docx]

## **Supplementary Table 1: Thresholds for seroprotection**

^WHO-recommended cutoffs

*Pertussis antibody concentrations, specifically against pertussis toxin, were measured using the international pertussis serum standard 10/142. As there is currently **no internationally recognised seroprotection cutoff for pertussis,** a threshold of 0.8 IU/ml corresponding to the lower limit of detection of the assay was used to indicate detectable antibody presence rather than confirmed protective immunity. A threshold of >100IU/mL was used to indicate recent or current pertussis infection.

**Supplementary Table 2: Demographic characteristics of participants**

**Supplementary Figure 1: Diphtheria-tetanus-pertussis (DTP) and measles-rubella (MR) vaccination status (n=379)**

**Supplementary Table 3: Seroprotection against five vaccine preventable diseases by vaccination status (n/%)**

Please note that vaccination data were available for 346/379 children (91%). Parents of an additional 28 individuals recalled vaccination of their children, but as dates could not be provided, these children were assumed to be partially immunised only, unless parents confirmed their child had not received any vaccinations to date. Partial vaccination also includes record of only 1 MR dose or only 1 or 2 DTP doses.
